# Optic nerve-mediated modulation of temporally interfering electric fields for potential targeted retinal disease therapy: a computational modeling study

**DOI:** 10.3389/fnins.2024.1518488

**Published:** 2024-12-23

**Authors:** Meixuan Zhou, Xiaofan Su, Tianruo Guo, Tianyue Meng, Weilei Wu, Liqing Di, Liming Li, Heng Li, Xinyu Chai

**Affiliations:** ^1^School of Biomedical Engineering, Shanghai Jiao Tong University, Shanghai, China; ^2^Graduate School of Biomedical Engineering, University of New South Wales, Sydney, NSW, Australia; ^3^Department of Orthopedics, Shanghai Pudong Hospital, Fudan University Pudong Medical Center, Shanghai, China

**Keywords:** computational modeling, extraocular electrical stimulation, temporal interference, optic nerve, retinal diseases therapy

## Abstract

**Introduction:**

Traditional extraocular electrical stimulation typically produces diffuse electric fields across the retina, limiting the precision of targeted therapy. Temporally interfering (TI) electrical stimulation, an emerging approach, can generate convergent electric fields, providing advantages for targeted treatment of various eye conditions.

**Objective:**

Understanding how detailed structures of the retina, especially the optic nerve, affects electric fields can enhance the application of TI approach in retinal neurodegenerative and vascular diseases, an essential aspect that has been frequently neglected in previous researches.

**Methods:**

We developed an anatomically accurate multi-layer human eye model, incorporating the optic nerve segment and setting it apart from current research endeavors. Based on this model, we conducted in *silico* investigations to predict the influence of the optic nerve on spatial characteristics of the temporally interfering electric field (TIEF) generated by diverse electrode configurations.

**Results:**

Optic nerve directly influenced spatial distributions and modulation rules of TIEFs. It caused convergent areas to shift nasally or temporally in relation to return electrode positions, and further increased the axial anisotropy within the convergent TIEF. Furthermore, alterations in electrode positions and adjustments to current ratios among channels induced diverse spatial patterns of TIEFs within the macular region, the area surrounding the optic nerve, as well as peripheral retina.

**Conclusion:**

Our findings suggested that presence of the optic nerve necessitated the utilization of different modulating paradigms when employing TI strategy for targeted treatment of various retinal lesions. And also provided theoretical references for developing a novel retinal electrical stimulation therapeutic device based on TI technology.

## 1 Introduction

Retinal neurodegenerative and vascular diseases, such as retinitis pigmentosa (RP), age-related macular degeneration (AMD), and retinal artery occlusion (RAO), can cause irreversible damage to the retinal neural and vascular networks (Wright et al., 2010; Barresi et al., 2023; Newton and Megaw, 2020; Scott et al., 2020). These conditions lead to vision impairment or even blindness, affecting more than 200 million people worldwide (Sather 3rd et al., 2023; Al-Namaeh, 2021; World Health Organization, 2020).

Transcorneal electrical stimulation (TES) (Li et al., 2024; Battaglini et al., 2022) has proven to be an effective therapeutic approach for partially restoring visual functions in patients with retinal neurodegenerative and vascular diseases. This method achieves its effects through neuroprotection or enhanced retinal blood flow (de Rossi et al., 2020; Stett et al., 2023; Meral et al., 2022; Kahraman and Oner, 2020; Nagasato et al., 2023; Naycheva et al., 2013; Inomata et al., 2007; Wang et al., 2011; Kurimoto et al., 2010). TES has also been shown to improve visual function in patients with optic neuropathies, further supporting its therapeutic potential (Miura et al., 2023; Fujikado et al., 2006).

Currently, clinical TES devices primarily employ either a Dawson-Trick-Litzkow (DTL)-Plus electrode (Schatz et al., 2011; Wagner et al., 2023) or an electroretinography (ERG)-jet electrode (Xie et al., 2012) fixed on the eyelid or the corneal surface and a return electrode at the distal end to deliver microcurrent to the retina. The output current is typically a square wave pulse with specific amplitude, frequency, and pulse width (Nagasato et al., 2023; Stett et al., 2023). However, these TES strategies only feature a single electrode channel at one stimulation trial, which results in a diffuse distribution of the induced electric field in specific areas on the upper or peripheral field of the retina (Xie et al., 2011; Hernández-Sebastián et al., 2023). Consequently, these devices lack the precision required to target specific regions and cannot steer the electric field to different lesion sites caused by the progression of various retinal diseases (Shepherd et al., 2013; Varma et al., 2013; Pinna et al., 2023).

Temporally interfering (TI) electrical stimulation, achieved by delivering kilohertz sinusoidal stimulation across multiple electrode channels with small frequency differences, has emerged with its capacity to selectively activate local neurons in the hippocampus of the deep brain through a convergent temporally interfering electric field (TIEF), and the application of this technology highly depends on a deep comprehension of electric field distribution characteristics (Grossman et al., 2017; Zhu et al., 2023). Previously, we theoretically validated the potential of the TI strategy in generating spatially convergent and steerable TIEFs on the retinal surface in an idealized human eye model (Su et al., 2021). However, the retinal surface in that study was a spatially uniform region, neglecting intricate biological structures within the eye, such as the optic nerve. Related studies with retina (Lyu et al., 2020; Song et al., 2020) or head models (Lu et al., 2022; Lee et al., 2021) also regarded the retinal surface as an integrated tissue to assess the spatial distribution of electric fields generated by various stimulation strategies. The optic nerve is a collection of millions of ganglion cell axons that are wrapped in myelin sheaths, and it extends from the optic disc of the retina to the lateral geniculate nucleus (Selhorst and Chen, 2009). Several studies demonstrated that electrical stimulation of the optic nerve through a penetrating or cuff electrode could effectively evoke a neural response in the retinal or visual cortex (Brelén et al., 2010; Lu et al., 2013; Borda et al., 2022). As a crucial neural pathway connecting the eye and brain, the optic nerve, while transmitting visual information, also constitutes an important environmental factor for electric field propagation due to its physical structure and electrophysiological characteristics. The impedance of the optic nerve is a vital parameter that cannot be neglected when exploring the distribution of electric fields on the retinal surface. Since myelin sheaths are almost electrically insulating, there is a significant difference in electrical conductivities along axial and cross-sectional directions of the optic nerve (Gabriel et al., 1996). However, existing research has mainly concentrated on optimizing electrode configurations, adjusting stimulation parameters, and directly observing the responses of retinal neurons (Su et al., 2021; Song et al., 2024) while lacking in-depth exploration and precise modeling of the optic nerve. Consequently, our understanding of the actual electric field distribution on the retina during TI stimulation remains limited, posing difficulties in accurately predicting stimulation effects and optimizing paradigms.

It is reasonable to hypothesize that the optic nerve could modify spatial distribution and modulation patterns of the retinal electric fields, which might affect the clinical therapy of retinal diseases with diverse categories or progressions via electrical stimulation, especially the TI strategy. Therefore, this study aims to systematically investigate the optic nerve-mediated spatial patterns of TIEFs generated by TI stimulation in an eye model that more accurately reflects physiological and anatomical characteristics. Herein, we constructed a human eyeball model with biophysically detailed retinal layers, an optic nerve, and surrounding tissues and applied TI stimulation through multiple extraocular electrode channels. The spatial distribution and modulation characteristics of generated TIEFs on the retinal surface mediated by the optic nerve were systematically calculated under several electrode montages and output current ratios. Our results revealed that when applying the TI strategy for targeted therapy of diverse retinal lesions or optic neuropathies, the existence of the optic nerve necessitated the selection of modulating paradigms and provided an innovative perspective for developing novel retinal electrical stimulation devices.

## 2 Materials and methods

### 2.1 Human eye model with optic nerve segment

**Figure 1:**
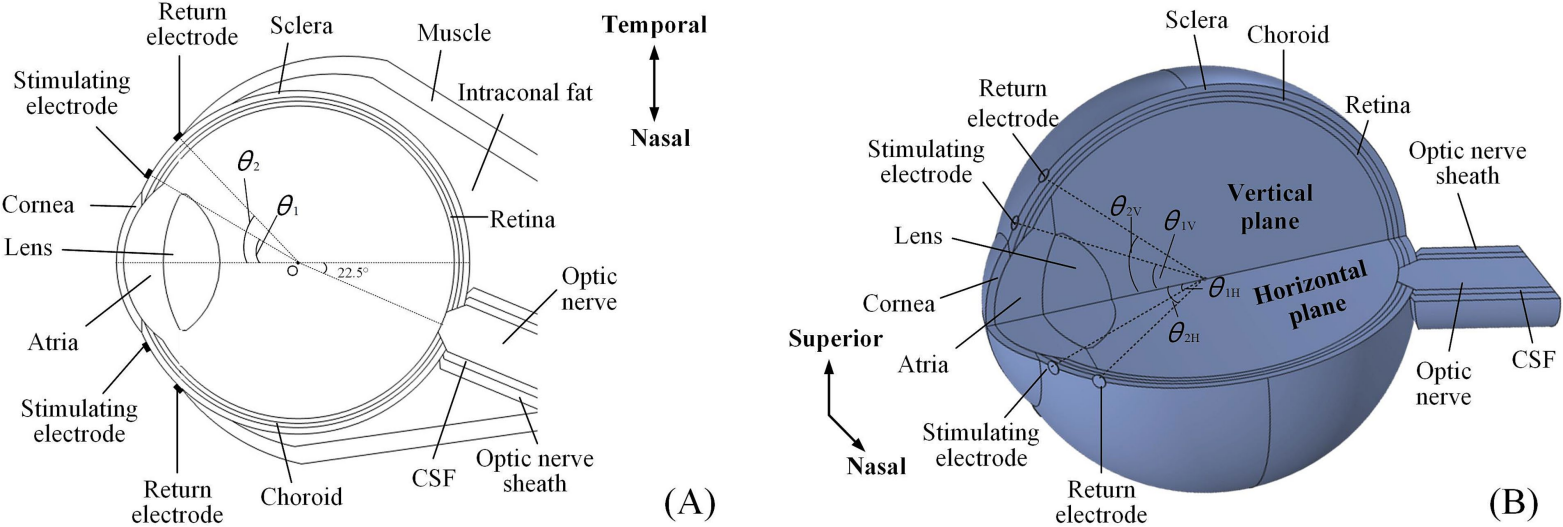
An anatomically detailed, multi-layer model of the human eyeball. **(A)** The 2D electrode montage featured four electrodes fixed symmetrically on the horizontal section of the model. **(B)** The 3D electrode channel montage comprised eight electrodes (a total of four channels) symmetrically distributed on both the horizontal and vertical planes, only showing superior and nasal channels.

A multi-conductivity 3D human eye model, incorporating essential structures such as the cornea, atria, lens, vitreous body (VB), retina, choroid, and sclera (Lyu et al., 2020; Cvetkovic et al., 2006), was constructed using the AC/DC module in COMSOL Multiphysics (Figure 1).

Additionally, the model also incorporated additional biological components, including the optic nerve, cerebrospinal fluid (CSF), the optic nerve sheath on the nasal side, and surrounding tissues such as muscle and fat.

Specifically, the optic nerve segment was positioned at a 22.5° angle relative to the model’s central axis, oriented horizontally toward the nasal side. The section intersecting the eyeball resembled a truncated cone, while the extended portion was cylindrical. Eventually, the optic nerve, CSF, and optic nerve sheath were aligned as coaxial cylinders. Based on reported data, the anterior segment of intraconal fat and muscle was shaped like hemispheres, while the posterior segment resembled a cone deviated from the optic nerve (Wang et al., 2016; Xu et al., 2017; Alireza et al., 2018). A single extraocular electrode was composed of a platinum (Pt) disc, measuring 500 μm in diameter and 50 μm in thickness, which was used to form extraocular multi-channel electrode montages for TI stimulation.

Table 1 outlines the conductivities of all tissues and Pt electrodes at 100 Hz, which were isotropic, except for the optic nerve. Given that the optic nerve was not perfectly aligned with the central axis of the eye, causing a 22.5° shift to the nasal side, the conductivity parameters listed in Table 1 assumed that the optic nerve was independent and aligned with the central axis. Therefore, the conductivity of the optic nerve was adjusted to account for this offset angle for ease of understanding.

**Table 1:** Conductivities and geometric parameters of eye model with an optic nerve segment.

Tissue/Body fluid/Electrode	Conductivity (S/m)	Thickness (mm)	References
Cornea	0.422	0.5	Song et al. (2020), Gabriel et al. (1996), Cela (2010), Oozeer et al. (2005), Cvetkovic et al. (2006), Wang et al. (2016), Stitzel et al. (2002), Won et al. (2016), Norman et al. (2011), and Tsukitome et al. (2015)
Atria	1.5	2.8	
Lens	0.322	4.0	
Vitreous body (VB)	1.5	22*	
Retina	0.5028	0.33	
Muscle	0.267	1.42	
Fat	0.028	14.5	
Choroid	0.2779	0.45*	
Sclera	0.5028	0.63	
Optic nerve	0.5 along the z (axial) direction; 0.08 along the x and y directions	3.55*	
Cerebrospinal fluid (CSF)	2	0.58	
Optic nerve sheath	0.006	0.73	
Platinum electrode	8.9 × 10^6^	-	

*The thickness of VB, fat, and optic nerve represent maximum diameters.

### 2.2 Electrode montages and current ratio indexes

We initially employed multi-channel electrode montages based on our eye model to analyze the spatial distribution of generated TIEFs through extraocular TI stimulation.

The 2D montage (Figure 1A) included two stimulating-return electrode pairs positioned horizontally around the eye and symmetrically aligned with the model’s central axis.

The specific positions of the stimulating and return electrodes were defined by the angles (*θ*_1_ and *θ*_2_, respectively) created between the lines connecting their centers to the eye’s center (point O) and the central axis of the asymmetric eye model. The 3D montage introduced another two symmetrical electrode channels around the vertical plane of the eyeball model (Figure 1B). In this setup, the stimulating and return electrodes on the horizontal plane were designated as *θ*_1H_ and *θ*_2H_, while those on the vertical channels were labeled as *θ*_1V_ and *θ*_2V_, respectively.

Furthermore, we defined current ratio indexes (illustrated as Equations 1, 2) as follows to explore the modulation principles of the TIEF on the retinal surface mediated by the existence of the optic nerve:


(1) $$\alpha_{X}=I_{1}/\left(I_{1}+I_{2}\right)$$

(2) $$\alpha_{Y}=I_{3}/\left(I_{3}+I_{4}\right)$$

Here, $\alpha_{X}$ represented the ratio of output currents from the nasal ($I_{1}$) and temporal ($I_{2}$) side of the horizontal stimulating electrodes, its value ranged from 0 to 1. Similarly, $\alpha_{Y}$ expressed the current ratio from stimulating channels on the superior ($I_{3}$) and inferior ($I_{4}$) sides of the vertical plane. The condition $\alpha_{X}/\alpha_{Y}=0.5$ represented equal current amplitudes across all channels.

### 2.3 TIEF calculation and analyses

Our previous study comprehensively described the basic calculating principles of the generated TIEF (Su et al., 2021). Briefly, a unit vector $\vec{n}$ represented any direction in space, and a 3D Cartesian coordinate system was utilized to pinpoint the spatial position of the envelope modulation amplitude resulting from the superposition of the $\vec{n}$ direction generated by multi-channel current sources at the position $\vec{r}\left(x,y,z\right)$ could be denoted as ${\vec{E}}_{\mathrm{AM}}\left(\vec{n},\vec{r}\right)$, which emerged as the vector sum of electric fields generated by two dependent current sources ${\vec{E}}_{1}\left(\vec{r}\right)$ and ${\vec{E}}_{2}\left(\vec{r}\right)$. Therefore, the envelope amplitude that yields TIEF at a specific point within the eye model was given by Equation 3 (Grossman et al., 2017):


(3) $$\left|{\vec{E}}_{\mathrm{AM}}\left(\vec{n},\vec{r}\right)\right|=\left|\left|\left({\vec{E}}_{1}\left(\vec{r}\right)+{\vec{E}}_{2}\left(\vec{r}\right)\right)\cdot \vec{n}\right|-\left|\left({\vec{E}}_{1}\left(\vec{r}\right)-{\vec{E}}_{2}\left(\vec{r}\right)\right)\cdot \vec{n}\right|\right|$$

As the electric field was vectorial, the maximum amplitude of its directional component at the position $\vec{r}\left(x,y,z\right)$ could be expressed as$\left|{\vec{E}}_{\mathrm{AM}-\mathrm{MAX}}\left(\vec{r}\right)\right|$, which was also the maximal modulated envelope amplitude (MMEA) of the TIEF at this location. Therefore, the MMEA of the 2D electrode montage could be described as Equation 4:


(4) $$\begin{array}{l} \left|{\vec{E}}_{\mathrm{AM}-\mathrm{MAX}}\left(\vec{r}\right)\right|=\\ \max\left\{\left|{\vec{E}}_{\mathrm{AM}}\left(\vec{n_{1}},\vec{r}\right)\right|,\left|{\vec{E}}_{\mathrm{AM}}\left(\vec{n_{2}},\vec{r}\right)\right|,\ldots ,\left|{\vec{E}}_{\mathrm{AM}}\left(\vec{n_{n}},\vec{r}\right)\right|\right\} \end{array}$$

Under 3D montage, as the incorporation of electrode channels, the MMEA of the generated TIEF at each point could be approximately calculated by Equation 5 (Grossman et al., 2017):


(5) $$\left|{\vec{E}}_{\mathrm{AM}}\left(\vec{n},\vec{r}\right)\right|=2\cdot \min\left\{{\vec{E}}_{1}\left(\vec{r}\right),{\vec{E}}_{2}\left(\vec{r}\right),{\vec{E}}_{3}\left(\vec{r}\right),\ldots ,{\vec{E}}_{n}\left(\vec{r}\right)\right\}$$

**Figure 2:**
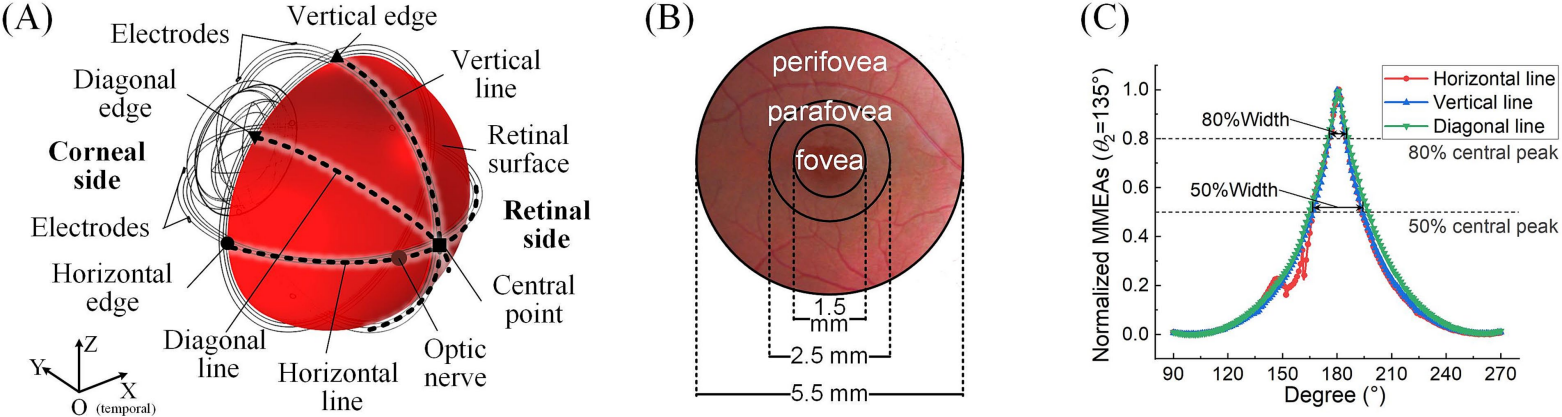
Definitions of retinal surface and calculation parameters. **(A)** Horizontal, vertical, and diagonal line centers and edge points on the retinal surface. **(B)** The fovea, parafovea, and peripheral fovea in the macular area. **(C)** FWHM and 80% peak width of the maximum-minimum normalized MMEA distribution curve along different directions (take *θ*_1_ = 30°, *θ*_2_ = 135°, and 3D electrode montage, for example).

For the analysis of TIEF’s spatial properties, we first calculated normalized MMEA values on the posterior retinal surface (red area in Figure 2A) and within the macula (Figure 2B) and further fitted MMEA curves to visualize the spatial distribution of generated TIEFs mediated by the optic nerve along horizontal, vertical, or diagonal directions (blacked dotted lines in Figure 2A). Moreover, we calculated the full width at half maxima (FWHM) and the full width at 80% maxima (80% width) of MMEA distribution curves to describe the spatial resolution of generated convergent TIEFs (Figure 2C), which physiologically represented excitation areas on the retinal surface under 2- and 1.25-times threshold stimulation intensities, respectively (Su et al., 2021).

Additionally, the peak values of retinal MMEA at the horizontal, vertical, diagonal edges, and center points were calculated and fitted to allow us to accurately compare variations of generated TIEFs under different simulation conditions. Finally, we determined the 80% intensity contour range and central peak offsets of different directional MMEA curves on the retinal surface and macula to further evaluate modulation patterns of TIEFs among different electrode montages and current ratios and reveal alternations of convergence and steerability mediated by the optic nerve.

## 3 Results

### 3.1 The optic nerve’s impacts on the spatial distribution of generated TIEFs

Initially, we demonstrated that altering the location of stimulating electrodes had minimal impact on the spatial patterns of the generated TIEFs when the optic nerve was present (Supplementary Figure S1). To ensure electrical safety, reduce potential invasiveness, and simplify implantation for clinical application, we fixed the stimulating electrodes at *θ*_1_ = 30° for all subsequent studies, approximately aligning with the electrode position employed in applied TES research (Xie et al., 2012).

**Figure 3:**
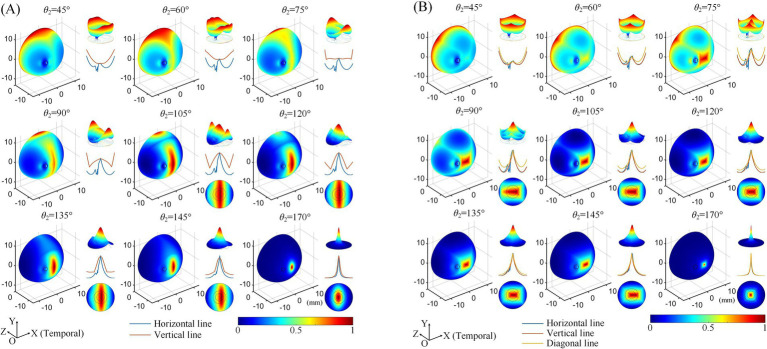
Normalized MMEA distributions of the retinal surface at different return electrode Positions using 2D **(A)** and 3D **(B)** electrode montages, *θ*_1_ = 30°, *θ*_2_ = 45°–170° (*θ*_2H_ = *θ*_2V_). Black dashed circle: cross-section of the optic nerve. The upper right of each illustration: a 3D mesh surface plot of the TIEF distribution of the retinal surface projected to the XOZ plane; middle right: MMEA values along the normalized horizontal and vertical lines; lower right: TIEF distribution in the macular area.

Subsequently, we explored the effects of varying the positions of the return electrodes, revealing trends consistent with our previous findings (Su et al., 2021). Regardless of whether a 2D (Figure 3A) or 3D (Figure 3B) electrode montage was used, as *θ*_2_ increased (with return electrodes moving closer to the retinal center), the strongest TIEF shifted from the peripheral edge to the retinal center. When *θ*_2_ ≥ 105°, a convergent electric field emerged in the central retina, with its range narrowing as the return electrodes approached the posterior eye. Additionally, the 3D montage improved the convergence of generated TIEF along the vertical axis, which was less pronounced compared to the horizontal axis, forming an approximately elliptical convergent area under the 2D montage.

However, since the optic nerve cross-section was on the nasal side of the retinal surface, an increased axial anisotropy emerged within the convergent TIEF. The most notable phenomenon was the sudden alternation observed in the MMEA distribution curve along the horizontal line for most electrode positions that could generate TIEFs with convergence (blue curve of each inset of Figure 3, except for *θ*_2_ = 170°). Additionally, the center of the convergent area shifted either temporally or nasally, depending on the relative position of the return electrodes to the optic nerve segment (*θ*_2_ ≤ 145° or = 170°). Moreover, compared to the square-shaped convergent TIEFs observed under all electrode positions in previous research (Su et al., 2021), the shapes of the TIEFs turned into near-rectangles when *θ*_2_ ≤ 145° in this study.

**Figure 4:**
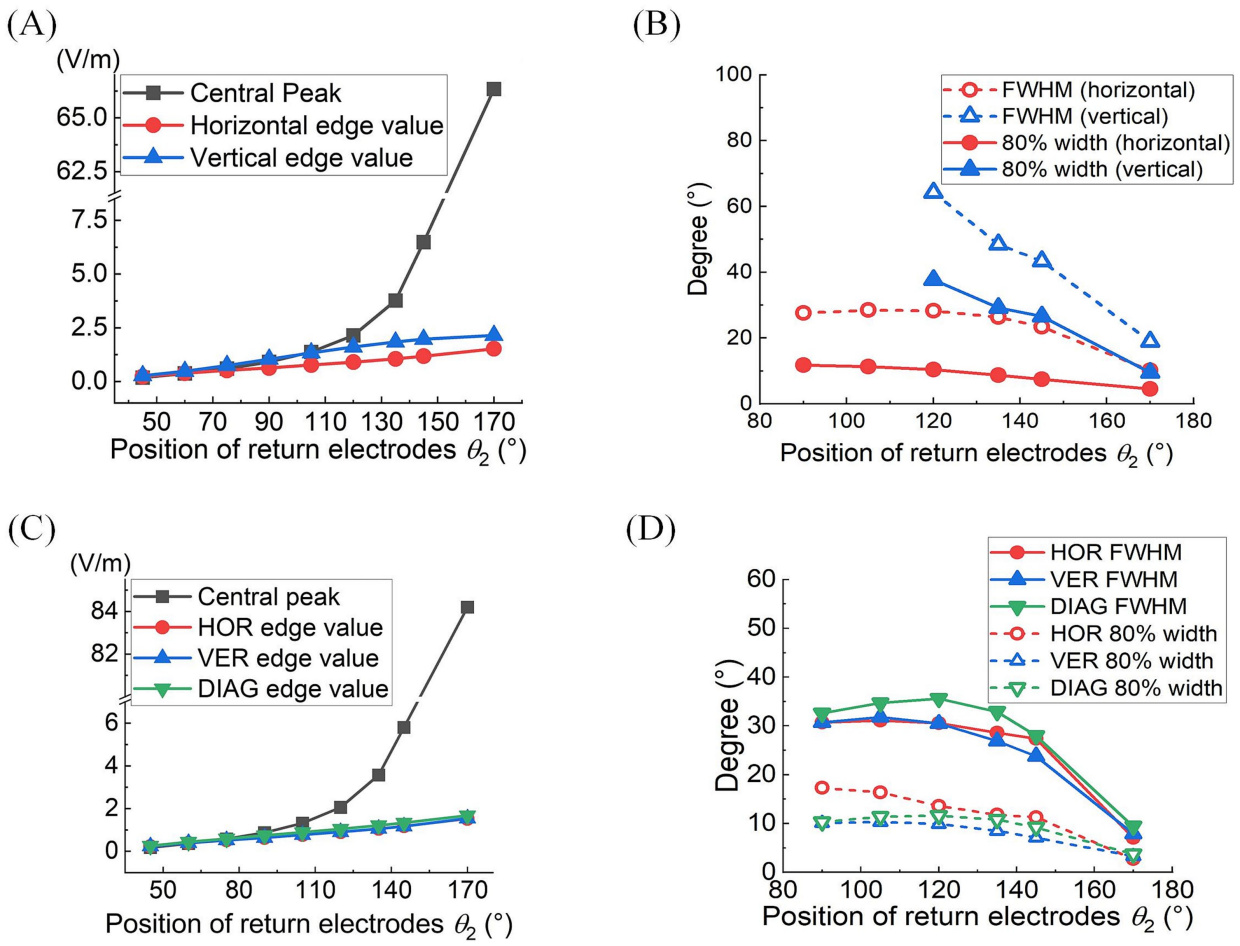
At different return electrode positions under 2D **(A,B)** or 3D **(C,D)** montage was employed: **(A,C)** MMEA values and fitting results of retinal center and edge points (*θ*_1_ = 30°, *θ*_2_ = 45°–170°). **(B,D)** FWHM and 80% widths of MMEA distribution curves along horizontal, vertical, and diagonal [only in **(D)**] lines of the retina (*θ*_1_ = 30°, *θ*_2_ = 90°–170°).

Quantitative analyses of MMEA properties indicated that as return electrodes progressively moved toward the posterior part of the eye, MMEA values at the retinal central point increased at a faster rate compared to those derived from horizontal, vertical, and edge points under both 2D and 3D montages (Figures 4A,C).

Specifically, when *θ*_2_ exceeded 105°, the MMEA at the central point gradually surpassed the edge values, indicating the generation of an increasingly intense electric field with a convergent peak in the central retina.

Figures 4B,D further illustrate the influence of return electrode positions on the TIEF’s spatial resolution. Under the 2D montage, while more posterior return electrodes improved the spatial resolution of the generated TIEF along both axes, the FWHM and 80% width of the horizontal line were always smaller than those of the vertical line, which was consistent with the elliptical convergent area displayed in Figure 3A. Another noteworthy finding was that the FWHMs and 80% widths of horizontal MMEA distribution curves were not always equal to those calculated from the vertical lines under the 3D montage (red and blue solid and hollow triangles in Figure 4D). This discrepancy further confirmed the anisotropy between the horizontal and vertical axes, as displayed in Figure 3B.

**Figure 5:**
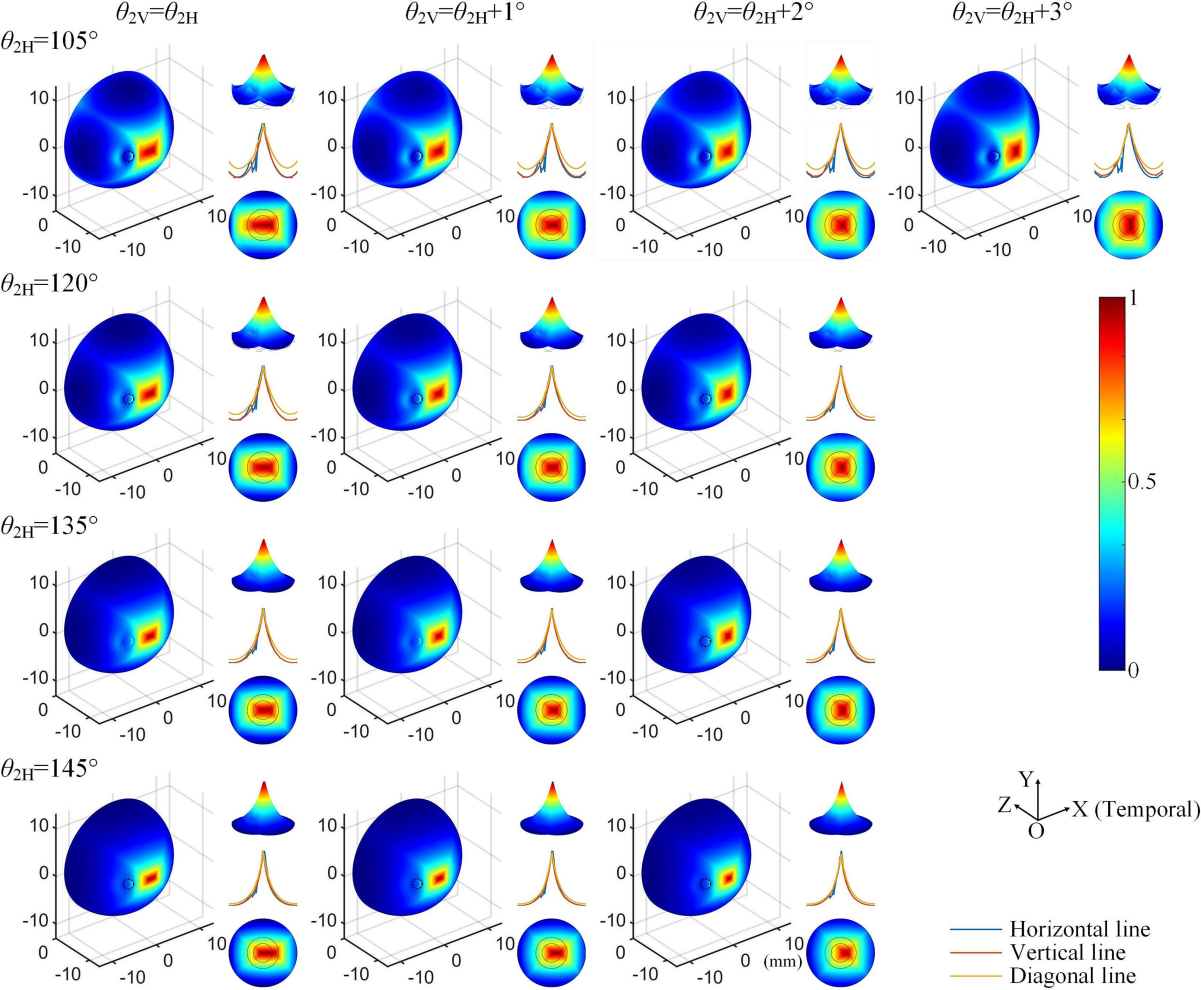
Normalized MMEA distributions (*θ*_1_ = 30°, *θ*_2_ = 105°–145°) of the retinal surface with adjustment of vertical electrode positions under 3D montage.

Considering the increased axial anisotropy mediated by the optic nerve to the convergent TIEF generated under initial current conditions ($\alpha_{X}/\alpha_{Y}=0.5$), as well as the asymmetry that would elevate its modulation complexity confirmed by our previous study (Su et al., 2021). We attempted to balance the horizontal and vertical convergence of the TIEF generated under 3D montages with return electrode positions ranging from 105° to 145° (the convergent area generated by *θ*_2_ = 170° maintained an almost square form and necessitated no adjustment). Figure 5 illustrated corresponding TIEF distributions, while Table 2 summarized resulting FWHMs and 80% widths of MMEA distribution curves along both axes, following alternations to the vertical electrode positions. As values marked by red boxes in Table 2, which demonstrated that TIEF generated under related electrode position could possess an optimal convergence accompanied by comparable axial isotropy, we finally determined *θ*_2H_ = 105° and *θ*_2V_ = 107° to represent *θ*_2_ = 105°, *θ*_2H_ = 120° and *θ*_2V_ = 121° to represent *θ*_2_ = 120°, *θ*_2H_ = 135° and *θ*_2V_ = 136° for *θ*_2_ = 135°, *θ*_2H_ = 145°, and *θ*_2V_ = 146° for *θ*_2_ = 145°, respectively.

**Table 2:** Alternations of 80% width and FWHM values along horizontal and vertical MMEA distribution curves after adjustments of return electrode positions under 3D electrode montage.

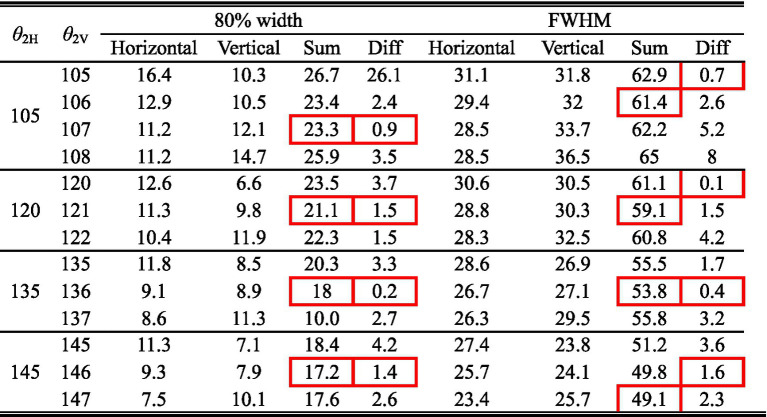

*Sum/Diff: summation/difference of the 80% width and FWHM along horizontal and vertical directions.

### 3.2 Modulation patterns of generated TIEFs on the retinal surface mediated by the optic nerve

**Figure 6:**
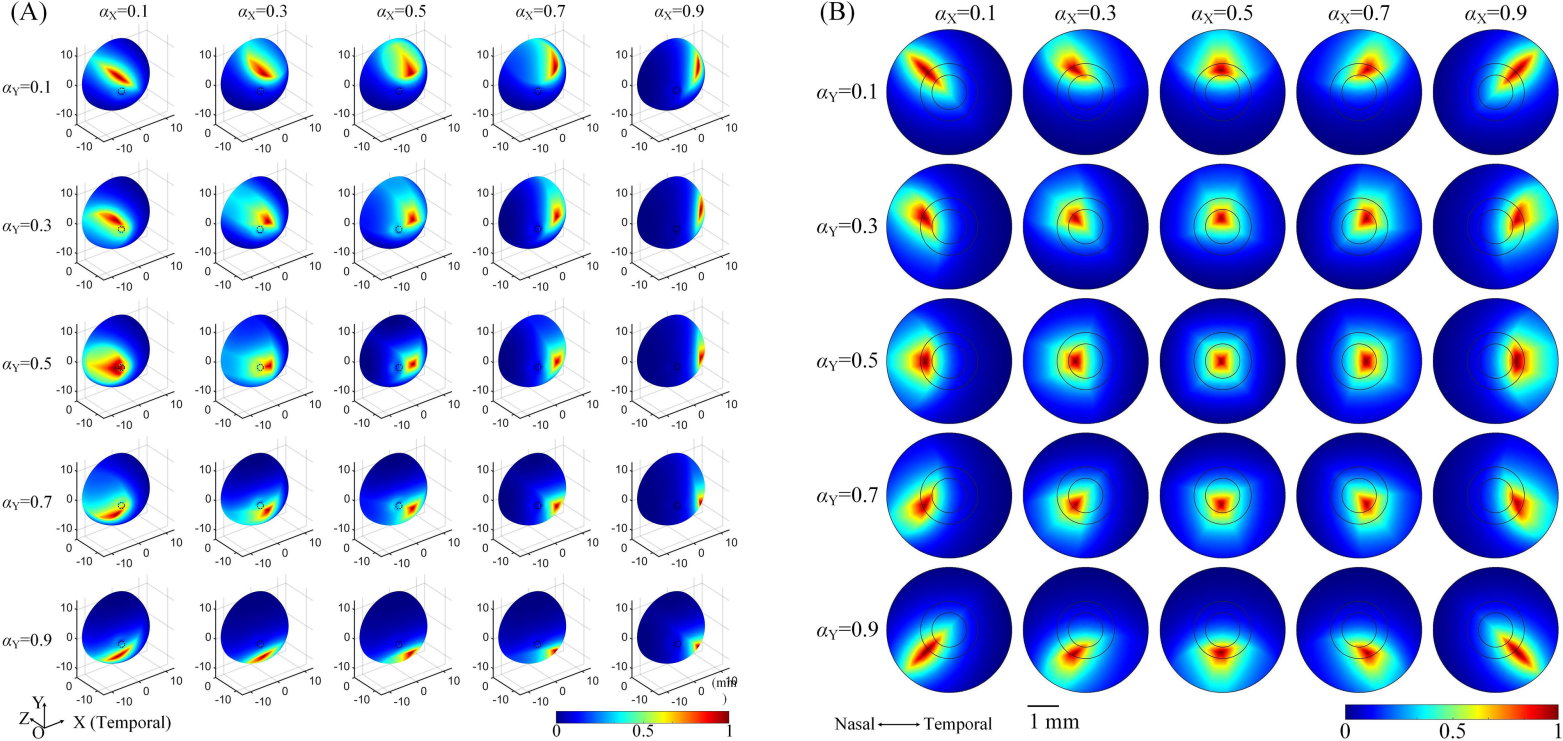
The normalized MMEA distribution of the retinal surface **(A)**, *θ*_2_ = 135° or macular area **(B)**, *θ*_2_ = 170° under different current ratios. $\alpha_{X}$ and $\alpha_{Y}$ = 0.1, 0.3, 0.5, 0.7, and 0.9, respectively. The specific location of the horizontal electrodes was slightly adjusted (referred to in Table 2 and Figure 5) to ensure the convergent TIEF was near square before steered by changing the current ratios ($\alpha_{X}$ and $\alpha_{Y}$ = 0.5).

The influence of the optic nerve on the steerability of convergent TIEFs was investigated by changing current ratios of horizontal ($\alpha_{X}$) and vertical ($\alpha_{Y}$) electrode channels under 3D montage and displaying modulation patterns on the retinal surface and within the macular area (Figure 6).

Taking the return electrodes fixed at *θ*_2_ = 135° as an example (illustrated in Figure 6A, *θ*_2H_ = 135°, *θ*_2V_ = 136°), the convergent area shifted on the retinal surface with alterations in $\alpha_{X}$ or $\alpha_{Y}$, or both simultaneously. Specifically, decreasing $\alpha_{Y}$ caused the convergent TIEF to gradually move to the superior side of the retinal surface and vice versa. Similarly, smaller/larger $\alpha_{X}$ resulted in the convergent region slowly shifting to the nasal/temporal side. Concurrent alternations of both $\alpha_{X}$ and $\alpha_{Y}$ could theoretically steer the generated TIEF across the entire retinal surface, even covering the full extent of the optic nerve cross-section. Modulation patterns under other return electrode positions exhibited similar tendencies, as displayed in Supplementary Figure S2. The steering behavior of the convergent TIEF around the macular area is shown in Figure 6B, where return electrodes were fixed at *θ*_2_ = 170°. Unlike the pattern presented in Figure 6A, the convergent area remained confined to the macula across all current ratios and exhibited a relatively regular shape, even when steered away from the retinal center.

**Figure 7:**
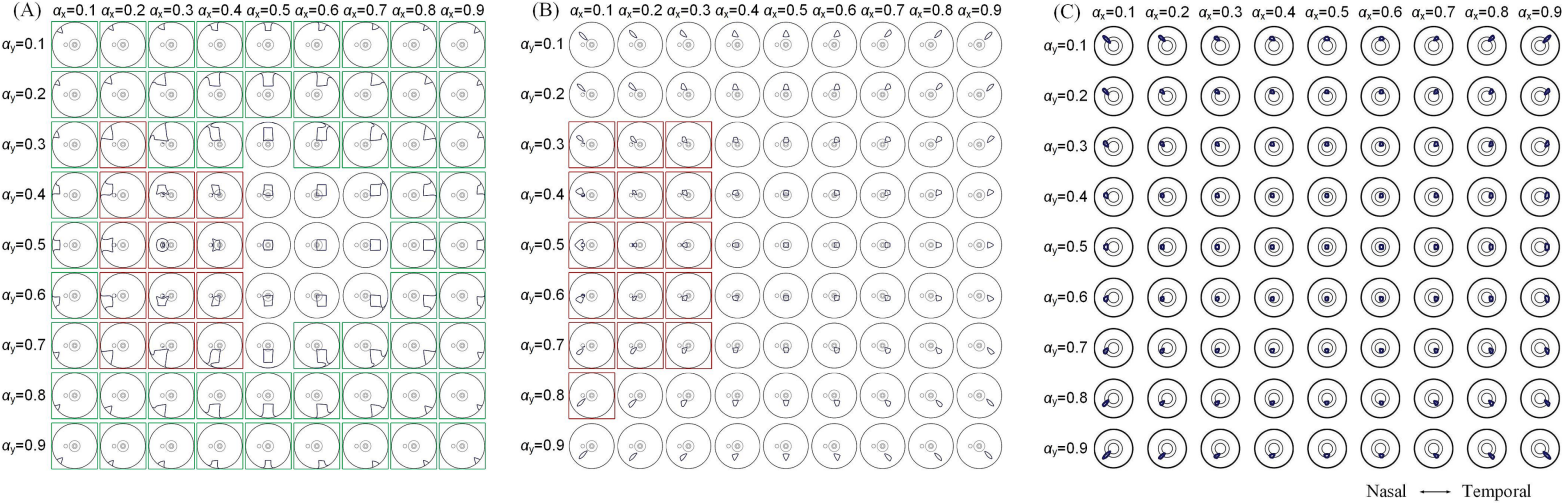
80% intensity contour ranges (dark blue lines) of normalized MMEA: on the retinal surface **(A,B)**, *θ*_2_ = 105° and 135°, and macular aera **(C)**, *θ*_2_ = 170° under different current ratios $\alpha_{X}$ and $\alpha_{Y}$ increased by 0.1 were projected to the XOZ plane, *θ*_1_ = 30°; green boxes: unclosed contour ranges on the retinal surface, red boxes: contour ranges covered optic nerve cross-section.

To further explore modulation diversities of the generated TIEF on the retinal surface mediated by the optic nerve, we calculated 80% intensity contour ranges of normalized MMEAs and presented three typical and distinct patterns in Figure 7 (*θ*_2_ = 105°, 135°, and 170°, respectively). Contour ranges assessed under *θ*_2_ = 120° and 145°were shown in Supplementary Figure S3. When return electrodes were fixed relatively close to the anterior retina (Figure 7A), alternations in current ratios resulted in a majority of the 80% intensity contours on the retinal surface remaining unclosed (marked by green boxes); additionally, some contours overlapped partially or completely with the optic nerve cross-section (red boxes), while a few could be modulated in the vicinity of the macula, displaying a convergent pattern. As return electrodes approached the posterior eye (Figure 7B), under various combinations of current ratios, generated TIEFs maintained convergent areas both in the central and peripheral retina. Notably, only in a few instances did the TIEFs partially or completely overlap with the optic nerve (marked by red boxes). While return electrodes were located surrounding the macula (Figure 7C), all generated TIEF contours exhibited a convergent pattern and were confined to the perifovea region; nevertheless, the degree of freedom in steering the TIEF contours with the macula was constrained by adjustments to the current ratios among electrode channels.

**Figure 8:**
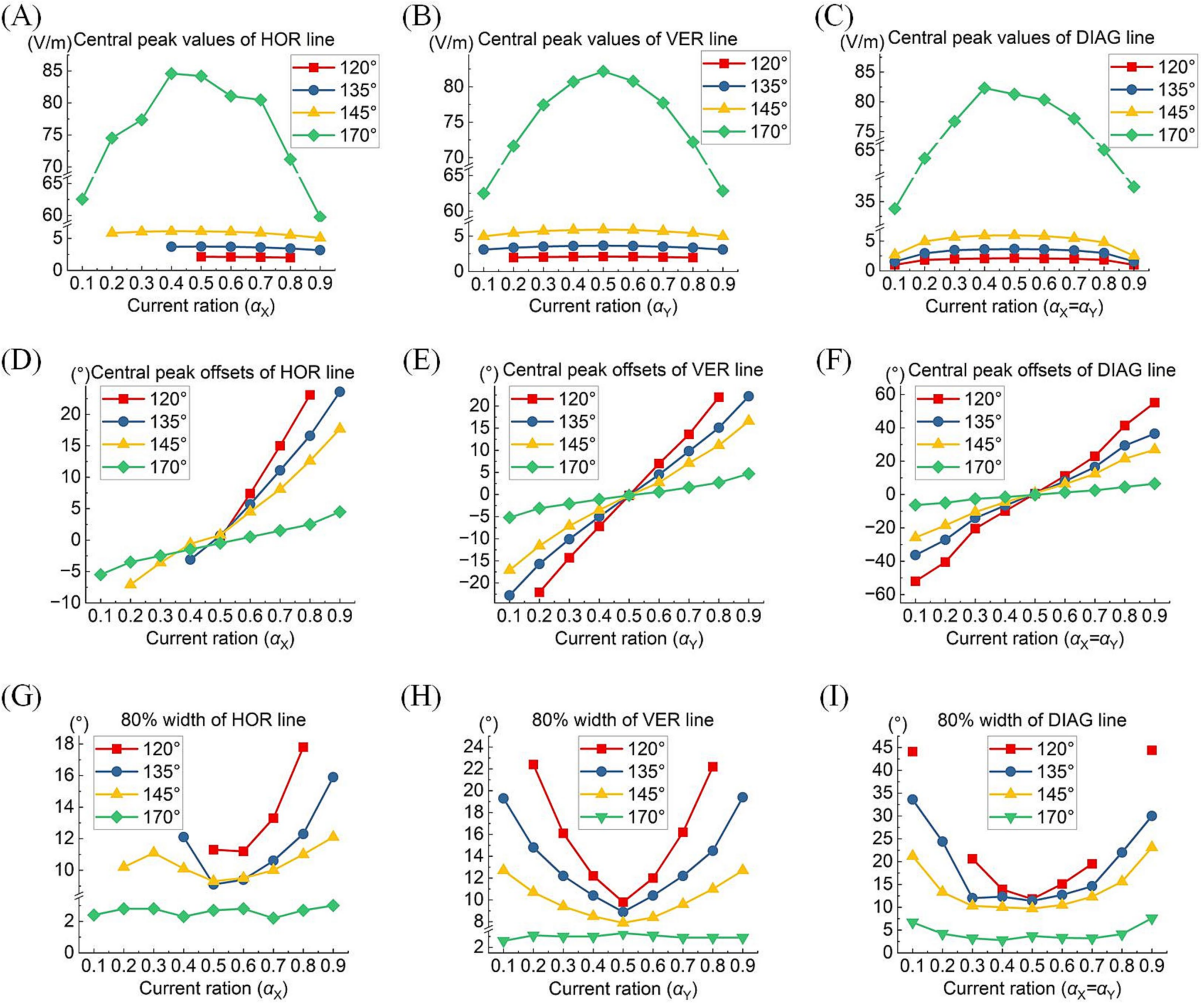
Central peak values (top row), central peak offsets (medium row), and 80% width (bottom row) of the MMEA distribution curves: along the horizontal line **(A,D,G)**, vertical line **(B,E,H)**, and diagonal line **(C,F,I)** of the retinal surface under different return electrode positions and current ratios (*θ*_2_ = 120° 135°, 145,° and 170°, TIEFs with unclosed contour ranges and covered optic nerve cross-section were excluded).

A comprehensive quantitative exploration was undertaken to investigate the modulation rules of TIEFs generated by different electrode positions and current ratios under the mediation of the optic nerve. We calculated central peak values and central peak offsets of MMEA curves in horizontal, vertical, and diagonal directions relative to the retinal center, along with their 80% peak widths in Figure 8 and Table 3. It is worth noting that these indices primarily focus on describing the spatial characteristics of closed and convergent TIEFs formed on the retinal area by altering current ratios. Therefore, we excluded conditions that generated a major proportion of unclosed TIEF (such as *θ*_2_ = 105°) or overlapped with the cross-section of the optic nerve (marked by red boxes in Figure 7).

**Table 3:** Modulation ranges of steerability and convergence of generated TIEFs on the retinal surface.

*θ*_2_	Central peak offset range			80% width of the TIEF distribution curves		
	Horizontal	Vertical	Diagonal	Horizontal	Vertical	Diagonal
120°	0 ~ 23°	±22°	±55°	14.5 ± 3.3°	16.1 ± 6.3°	28.1 ± 16.3°
135°	-3° ~ 23.6°	±23°	±36.5°	12.5 ± 3.4°	14.2 ± 5.3°	22.5 ± 11.1°
145°	−7° ~ 17.7°	±17°	±27°	10.7 ± 1.4°	10.3 ± 2.4°	16.4 ± 6.7°
170°	−5.5° ~ 4.5°	±5°	±6.5°	2.6 ± 0.4°	3 ± 0.4°	5.2 ± 0.4°

Typically, the largest central peaks were observed when *θ*_2_ = 170° and the current ratio was 1:1 (i.e., $\alpha_{X}$ = $\alpha_{Y}$). These peaks varied significantly as the convergent TIEF moved in all directions across the retinal surface (green lines in Figures 8A–C); however, they remained relatively stable under other return electrode montages. As shown in Figures 8D–F, changing $\alpha_{X}$ or $\alpha_{Y}$ steered the generated TIEF from the central to the peripheral retinal surface, which led to a negative (nasal side) or positive (temporal side) increase in central peak offsets. Due to the presence of the optic nerve, the offset ranges under most return electrode positions (except *θ*_2_ = 170°) along the horizontal direction (Figure 8D) were anisotropic and predominantly confined to the temporal sides of the optic nerve cross-section.

Moreover, although the steerable range of the convergent area narrowed as return electrodes approached the posterior eye, its convergence presented more predominantly (the absolute values of 80% widths were smaller in a larger return electrode position illustrated in Figures 8G–I). Theoretically, the optic nerve did not limit the steerable regions along vertical and diagonal directions (Figures 8B,E,F). Notably, a relatively high-intensity electric field might be generated in the peripheral retina or the optic nerve cross-section.

Table 3 summarizes the central peak offsets of the convergent TIEFs and the variation ranges of their 80% width (indicating convergence or spatial resolution) of the distribution curves along three directions. Briefly, the central points of convergent TIFFs generated and steered when *θ*_2_ ≤ 145° exhibited a similar horizontal offset range (roughly 25°) without crossing the optic nerve, and its range narrowed along vertical and diagonal directions with return electrodes moving posteriorly. The minimum 80% widths in different directions also decreased with larger *θ*_2_ values, indicating an improvement of the convergence or spatial resolution with a more posterior return electrode location. At *θ*_2_ = 170°, the convergent area under various steerable conditions was primarily limited in the parafovea region of the macula, with absolute offset ranges not exceeding 10° horizontally and vertically. The most convergent sites were generated in the fovea with an 80% width of less than 6° along all directions (last row in Table 3).

## 4 Discussion

Using a human eye model that incorporates detailed retinal structures, especially the optic nerve segment, our study confirmed that spatial characteristics of the temporally interfering electric field generated by the extraocular TI strategy were remarkably and diversely mediated by the presence of the optic nerve. Based on these new findings, we suggested that the electrode montages and stimulation parameters for TI stimulation should be flexibly configured to treat retinal diseases and optic neuropathies at different lesion locations or stages of progression.

### 4.1 Diverse distribution and modulation patterns of TIEF mediated by the optic nerve

In comparison to a previous study that used an idealized symmetrical eye model to simulate the retinal electric fields generated by the TI approach (Su et al., 2021), this study initially demonstrated similar trends in the convergence and steerability of the generated TIEF: (1) A convergent area was established when the return electrodes were positioned relatively close to the posterior side of the retina (*θ*_2_ > 105°); (2) the TIEF could be steered in the direction with smaller current ratios, regardless of whether the adjustment was made horizontally, vertically, or diagonally. However, incorporating more intricate biological tissues, such as the optic nerve segment, in this study introduced variations in the distribution and modulation patterns of the TIEF.

#### 4.1.1 The optic nerve caused the convergent TIEF and retina to no longer coincide with the center TIEFs, which no longer overlapped with the retinal center

**Figure 9:**
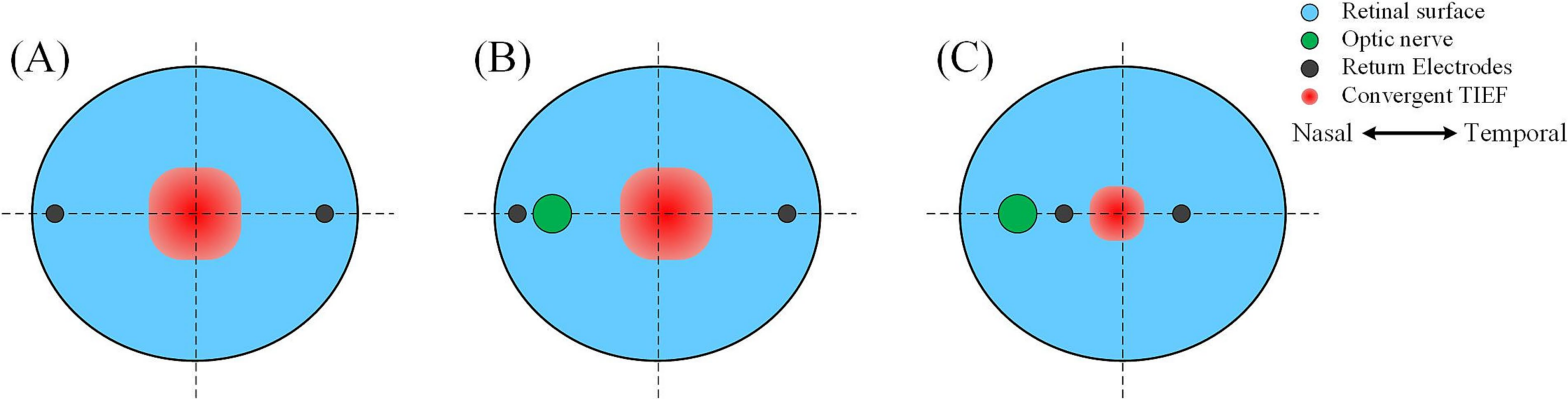
Effects of the relative position between return electrodes and the optic nerve on the distribution of convergent TIEF. **(A)** The idealized eye model was employed in the previous study, where one return electrode was located on the **(B)** temporal or **(C)** nasal side of the optic nerve.

Under both 2D and 3D electrode montages depicted in Figure 3 (lower right of each insert), the generated TIEF did not align with the retina’s center point (macular fovea) horizontally. Interestingly, we found that when horizontal electrodes “clamp” the optic nerve in the middle (i.e., *θ*_2_ = 105°, 120°, 135°, and 145°), the TIEF’s center shifted temporally. It seemed as if the optic nerve “pushed” it in the opposite direction (Figure 9B); conversely (*θ*_2_ = 170°), the convergent TIEF deviated nasally, as if the optic nerve “attracted” the electric field to its side (Figure 9C). In our previous idealized eye model, regardless of how return electrode positions changed, the center of the convergent TIEF almost strictly overlapped with the retinal center (Figure 9A).

Related research on transcranial electrical stimulation indicated that the intensity and distribution of the electric field were influenced by multiple factors, including electrode location, size, distance, and the physiological characteristics of the tissues (Shahid et al., 2014). In this study, because our eye model incorporated the optic nerve along the horizontal return electrodes path, there is a noticeable conductivity variation on the retinal surface at the optic nerve, which made the area of interest no longer an isotropic and homogenous tissue. However, the reason why the relative position between return electrodes and the optic nerve could induce two contrary results was still unknown. Limited by computational modeling capabilities, we could not delve into the more fundamental analysis, and future neuronal response or electromagnetism studies were crucial to reveal underlying physiological or physical mechanisms.

#### 4.1.2 Axial anisotropy of the convergent area increased since the existence of the optic nerve

Furthermore, we discovered that the presence of the optic nerve increased the directional anisotropy to the TIEF under the initial simulation conditions. As illustrated in Figure 3B, the shapes of all convergent areas appeared as rectangles, contrasting with the horizontally and vertically isotropic squares observed in our earlier research (Su et al., 2021). We hypothesized that the abrupt change in conductivity near the optic nerve segment on the retinal surface may exert a specific “pulling effect” on the TIEF in the horizontal direction (Gabriel et al., 1996). This phenomenon warrants further investigation to elucidate the precise mechanism involved.

Given that the retina was modeled as an electrically isotropic layer, it can be speculated that the therapeutic effects of electrical stimulation might be influenced by the increasing axial anisotropy of electric fields generated in biological tissues (Metwally et al., 2015; Howell and Mcintyre, 2016; Zhao et al., 2021). To address this, we adjusted the positions of the vertical return electrodes when *θ*_2_ < 170° (typically by 1–2 degrees, resulting in *θ*_2H_ ≠ *θ*_2V_) to maintain relative axial isotropy of the generated TIEF along horizontal and vertical directions under the initial simulation conditions (as shown in Table 2 and Figure 6).

#### 4.1.3 The optic nerve segment resulted in diverse modulation patterns in the retinal region

While the convergent TIEF could previously spread across the entire retinal surface with various current ratios (Su et al., 2021), the steerable electric fields exhibited three distinct patterns affected by the optic nerve. Specifically, when one horizontal return electrode is fixed at the nasal side of the optic nerve (*θ*_2_ < 170°), it could generate unclosed contours on the posterior retina while steering TIEFs to the peripheral regions under various current ratios. The possibility of this occurrence increased as return electrodes were fixed anteriorly (green boxes marked in Figure 7A; Supplementary Figure S3A). Meanwhile, TIEFs steered to the central retinal under these electrode positions remained a convergency among certain current ratios (central illustrations not marked by green or red boxes in Figures 7A,B). On the other hand, when horizontal return electrodes are located at the temporal side of the optic nerve, the generated TIEF can be precisely and freely steered within the macula while maintaining its convergence (as shown in Figure 7C). Moreover, in some specific instances, the optic nerve cross-section could be partially or entirely encompassed by a generated electric field featuring either convergent or diffuse contours (red boxes marked in Figures 7A,B). These findings suggested that if the primary object was to achieve selective activation of specific retinal regions through a convergent TIEF, the horizontal steering range was strictly confined to the temporal side of the optic nerve.

### 4.2 Feasibility of selective therapy for retinal neurodegenerative, vascular, and optic nerve diseases

Electrical stimulation has been widely applied to the therapy of various retinal diseases, including retinitis pigmentosa (RP), age-related macular degeneration (AMD), optic neuropathy (ON), and retinal artery occlusion (RAO)(Meral et al., 2022; Nagasato et al., 2023; Stett et al., 2023; Miura et al., 2023; Parkinson et al., 2023). Presently, micro-current stimulation is delivered via a single stimulating and return electrode channel through trans-corneal, palpebral, or orbital approaches, resulting in a diffuse electric field limited to specific retinal regions (Inomata et al., 2008; Sabel et al., 2021; Xie et al., 2012; Xie et al., 2011; Hernández-Sebastián et al., 2023). However, lesion locations differ among retinal diseases and may spread or change as the pathology progresses. For example, damage in early to mid-stage RP patients initially affects the peripheral retina. It gradually spreads to the central area, while in AMD, the lesion expands from the macula to the entire retina (Shepherd et al., 2013). Similarly, central and branch retinal artery occlusions (CRAO and BRAO) occur in the corresponding macula or peripheral region (Scott et al., 2020). Therefore, traditional single-channel electrical stimulation strategies may not realize selective therapy for local retinal lesions on a small scale.

Recent studies have demonstrated that combining multi-channel electrical stimulation with optimized current parameters can somewhat activate neurons in the posterior retina (Lee et al., 2021). Relative research also suggested that multi-channel stimulation offers advantages in selectively activating the local retina and generating specific stimulation patterns, potentially beneficial for treating central, peripheral, or entire visual field injuries (Hernández-Sebastián et al., 2023). In contrast, the present study adopted the TI strategy, which incorporated multiple extraocular stimulating-return electrode pairs and resulted in convergent electric fields under several electrode montages. Moreover, due to the consideration of the presence of the optic nerve, this strategy generated diverse electric field distribution and modulation patterns on the retinal surface. How to effectively leverage this diversity could offer the potential for the selective treatment of various retinal and optic nerve diseases.

#### 4.2.1 Further posterior return electrodes were demanded for the therapy of central retinal diseases through the TI strategy mediated by the existence of the optic nerve

To effectively treat retinal neurodegenerative and vascular lesions affecting the central visual field (i.e., AMD and CRAO), generating an electric field that converges in the macula with precise steerability is crucial. In this study, when both return electrodes were fixed on the temporal side of the optic nerve (Figure 3B), we observed an optimal convergent TIEF around the parafovea region. This kind of TIEF exhibited an 80% width of 2.6° and 3.0° (Table 3) along the horizontal and vertical axes, respectively, corresponding to an activated area of approximately 0.52 mm × 0.63 mm with 1.25 times threshold stimulation. This convergent area could be steered in a small range around the fovea and was unaffected by the optic nerve section (Figures 6B, 7C).

We also observed slight improvements in the FWHM values, reducing to approximately 8° horizontally and vertically (Figure 4D) compared to our previous research (approximately 10°) (Su et al., 2021). We hypothesize that this enhancement could be due to the change in the external environment from saline to muscle and fat in this asymmetric eye model, potentially confining the electric field within the internal eye structure (Cela, 2010).

For cases where lesions affect the entire macula, precise steering of the TIEF may not be necessary. A convergent TIEF covering the majority of the central retina, as shown in the central conditions displayed in Figures 7A,B, may be more suitable for diffuse therapy targeting related diseases. However, it is important to ensure that such a convergent TIEF is not freely steered, as this could lead to unintended activation in non-pathological areas, such as the retinal periphery or the optic nerve.

Notably, some retinal degenerations are concentrated in the central macula with a relatively small lesion region (Stone, 2007), so aligning the generated TIEF with the fovea from a clinical perspective is preferable. Although the axial anisotropy of the convergent TIEF was considerably eliminated by the alteration of the vertical return electrode (Figure 5), a horizontal deviation from the retinal center still emerged. Since two horizontal return electrodes were completely symmetrical about the vertical plane, slight adjustments to their relative positions (i.e., *θ*_2Nasal_ ≠ *θ*_2Temporal_) in future work might improve the alignment of the convergent TIEF with the macula. These findings further confirmed the feasibility of achieving convergent and targeted treatment for diseases affecting the central retina through our proposed electrical stimulation strategy.

#### 4.2.2 The existence of the optic nerve does not constrain the therapeutic range of peripheral retinal diseases under the TI strategy

We observed that despite certain electrode montages not being optimal for focal stimulation of the central retina, they still effectively generated relatively high-intensity electric fields in the peripheral region, including the perifovea area, even if the contours of the generated electric fields were not fully enclosed (Figures 3B, 5, 7A). Furthermore, the existence of the optic nerve appeared to minimally affect the spatial distributions of these convergent or diffuse TIEFs on the peripheral retinal surface, as with specific current ratios, their contour ranges were possible to “bypass” the optic nerve cross-section.

Additionally, peripheral retinal neurons are more sparsely distributed compared to those in the central region, and visual acuity tends to decline as eccentricity increases (Muniz et al., 2014; Low, 1951), which suggests that a broader area is required in the peripheral retina to activate an equivalent number of neurons. In the case of RP, early- to mid-stage patients exhibit lesions primarily affecting the peripheral visual field, which gradually progress toward the central region (Mrejen et al., 2017; Liu et al., 2022). As for BROA, only a portion of the retinal function is moderately impaired (Schmidt et al., 2020; Dattilo et al., 2018), suggesting that optimal convergence in the central retina is not the foremost consideration when seeking effective therapies for mild-stage neurodegenerative or vascular lesions in the peripheral retina. Therefore, various electrode montages proposed in our TI electrical stimulation method could be viable in clinical applications. For example, steerable conditions marked by green boxes in Figure 7A and Supplementary Figure S3A offer possibilities for peripheral therapy for patients with RP or BROA. Even if it might induce unclosed contours or irregular shapes, it remained feasible to generate regional electrical stimulation in the retinal areas corresponding to different quadrants of the visual field, disregarding the existence of the optic nerve.

#### 4.2.3 TI strategy may enable effective targeting therapy for optic neuropathy

During electrical stimulation treatment for retinal lesions, the optic nerve is typically not the intended target, as its activation will lead to a wide range of meaningless perceptions (Veraart et al., 2003), which can complicate the selection of appropriate stimulation intensities based on individual electrical-evoked phosphene thresholds (Kahraman and Oner, 2020; Meral et al., 2022; Wagner et al., 2023). Nonetheless, for specific optic neuropathies (Fujikado et al., 2006; Miura et al., 2023; Gall et al., 2016), electrical stimulation has proven therapeutically beneficial, inspiring us to discuss targeting treatments for optic neuropathy utilizing an extraocular TI strategy. Notably, a few modulation sites depicted in Figures 7A,B, and Supplementary Figure S3A (highlighted by red boxes) exactly covered the entire cross-section of the optic nerve, making them potential candidates for focal electrical stimulation therapy. However, steering these TIEF sites demands careful coordination between electrode montages and current ratios, posing challenges in defining electrode implantation procedures and stimulation parameters in clinical applications.

Regrettably, we mainly focus on the spatial characteristics of TIEFs generated on the retinal surface and fix electrode channels only on the extraocular surface, neglecting TIEFs along the optic nerve axis. It is conceivable that by positioning return electrodes further posterior in the eye, such as on the myelin sheath surface, it may result in more complex functional activations and generate a convergent TIEF with high intensity at varying depths along the optic nerve (Sakaguchi et al., 2009).

### 4.3 Promotion for the development of novel retinal electrical stimulation devices

Current TES devices primarily consisted of a stimulating electrode attached to the corneal surface, eyelids, or orbital skin, along with a return electrode located at the occipital pole, forearm, or thigh, and either an external or integrated microcurrent stimulator (de Rossi et al., 2020; Stett et al., 2023; Parkinson et al., 2023). The electric current reaches the retina through a minimally invasive or even non-invasive manner, with the most severe side effects being tolerable symptoms, such as mild inflammation and dry eyes, which are easily treatable (Schatz et al., 2017; Wagner et al., 2017).

In contrast, the extraocular TI stimulation proposed here involves a more invasive approach, as the return electrodes are implanted in the posterior eye (*θ*_2_ ≥ 105°). In practical applications, stimulating electrodes can be modified as contact lenses that are attached to the corneal surface (Xie et al., 2012). During the implantation of the return electrodes, minimally invasive surgery is necessary to incise the skin and muscular tissue at the corner of the eye (Soleymanzadeh et al., 2023), position the return electrodes at the appropriate location behind the eyeball, and subsequently suture it to the adjacent tissues. The wired connection between the electrodes and the external simulator can also hinder eye movement. However, this extraocular electrode implantation procedure requires no incision or destruction of the eyeball, nor does it harm the retina (Weiland and Humayun, 2014), enabling multiple repeated surgical implantations. We anticipate that advancements in novel electrode materials [such as carbon nanotube electrodes (Devi et al., 2021)] and wireless coding technologies will facilitate the implantation of multiple flexible electrode contacts with excellent conductivity and biocompatibility in the posterior eyeball simultaneously. The wireless return electrode array on the retinal surface will possess various spatial configurations, such as a concentric ring: the inner return electrodes can be fixed at the temporal side of the optic nerve cross-section, aiming for targeted and steerable stimulation of the macular area, while the outer ring is located at a more anterior position to achieve peripheral visual field or optic nerve therapy. Prior to each treatment session, the stimulating electrode array could be positioned on the corresponding corneal surface based on the lesion area and the corresponding return electrodes to generate a steerable TIEF through the TI strategy.

Besides, current TES devices are limited to generating a single current waveform at once, typically a charge-balanced rectangular pulse with a certain amplitude, phase duration, and frequency (Nagasato et al., 2023; Stett et al., 2023). The retina, however, is an intricate tissue composed of multiple neural layers (such as photoreceptors, bipolar, and ganglion cells), and each neuron type prefers a different frequency of electrical stimulation (Freeman et al., 2010; Hadjinicolaou et al., 2016). Traditional TES methods may not be able to selectively activate multiple impaired neurons in the lesion area simultaneously. Conversely, an innovative retinal electrical stimulation device equipped with multi-extraocular stimulation channels will have the potential to generate several TIEFs with distinct envelope frequencies, distributed either separately or concentratedly through the TI approach (Zhu et al., 2019).

### 4.4 Limitation

In this study, we conducted computational calculations to investigate the distribution patterns and modulation principles of TIEFs generated by extraocular TI stimulation in a biologically detailed human eye model. However, in practical applications, additional non-ocular structures, including the skull, skin, and even brain tissues, may influence the spatial characteristics of the TIEF (Lu et al., 2022; Lee et al., 2021), exemplifying the abrupt change in the MMEA curve around the optic nerve along the horizontal axis (middle illustrations in Figure 3). In the future, it is imperative to integrate our detailed eyeball model into a realistic human head model derived from medical imaging techniques, such as MRI (Cvetković et al., 2017; Haberbosch et al., 2019), which may provide more practical guidance for clinical electrode implantation.

Furthermore, the optic nerve serves as the crucial link between the eye and the brain, comprising millions of ganglion cell axons responsible for transmitting action potentials to the lateral geniculate nucleus (Selhorst and Chen, 2009). Several studies have demonstrated that electrical stimulation can partially restore visual functions compromised by optic neuropathy (Miura et al., 2023; Gall et al., 2016; Gall et al., 2010; Fedorov et al., 2011; Sabel et al., 2011; Bola et al., 2014). In our study, the extraocular TI electrical stimulation also exhibited a relatively convergent TIEF that covered the optic cross-section under certain strategies, but distributions of the generated TIEF along the axial direction of its depth remain unexplored.

Finally, our study mainly focused on spatial characteristics of simulated electrostatic fields, neglecting the potential neural responses evoked by the generated TIEF. For example, current intensity is a critical parameter that affects neuronal response and safety in assessing the effectiveness of electrical strategies in restoring visual function (Schatz et al., 2011; Schatz et al., 2017; Perin et al., 2019). Additionally, it has been suggested that the carrier frequency of TI stimulation should be high enough (− kHz) to elicit the desired neuronal response (Twyford et al., 2014; Esmaeilpour et al., 2021), while the envelope frequency should match the optimal response frequency of target neurons (5–100 Hz)(Freeman et al., 2010; Hadjinicolaou et al., 2016; Su et al., 2022). Related studies based on a population retinal ganglion cell (RGC) model demonstrated that when return electrodes were fixed in the macular (*θ*_2_ ≥ 170°), the threshold for activating RGC populations in response to TI stimulation was lower than 2 mA and increased with higher carrier frequency (Song et al., 2024), which indirectly confirmed that in our study when *θ*_2_ = 170° and $\alpha_{X}=\alpha_{y}=0.5$, the central peak value of the MMEA generated on the retinal surface was sufficient to elicit a neural response. For other electrode montages or steerable patterns, it might be appropriate to increase the current intensity of stimulating channels or apply electrodes with stronger charge injection capabilities, such as carbon nanotube electrodes (Devi et al., 2021). However, *in vitro* or *in vivo* experiments were still necessary to determine the current safety limits required to elicit robust retinal responses with minimal side effects and to investigate the characteristics of specific retinal neurons in response to different stimulation parameters. Furthermore, the physiological feasibility of TI strategy in visual function restoration should be verified in clinical trials.

## 5 Conclusion

Here, we constructed a multi-layer human eye model, incorporating the optic nerve segment and surrounding tissues, to explore the spatial characteristics of TIEFs influenced by the optic nerve using an extraocular TI stimulation approach. The results suggested that the existence of the optic nerve modified the distribution and modulation principles of TIEFs on the retinal surface generated by the TI strategy. It brought increased axial anisotropies to the convergent area related to the return electrode positions and induced diverse steerable patterns of the TIEF that emerged across various retinal regions, such as the macula, the area surrounding the optic nerve, and the peripheral retina.

Our results provided an innovative indication that the TI strategy was available for targeted therapy for different retinal lesions or optic neuropathies, emphasizing the need to consider the optic nerve when selecting modulation paradigms. These findings also established a theoretical framework for the development and implementation of innovative retinal electrical stimulation devices based on TI technology. Nevertheless, developing viable clinical protocols derived from this methodology and further animal and human trials are essential to validate the therapeutic benefits of TI stimulation for retinal and optic nerve diseases.

## Data Availability

The raw data supporting the conclusions of this article will be made available by the authors, without undue reservation.

## Supplementary material

SUPPLEMENTARY FIGURE S1 Spatial distribution of TIEFs under different stimulating electrodes positions. **(A)** Normalized MMEA distribution of the retinal surface at different return electrodes positions with 2D electrode montage (*θ*_1_ = 30°–120°, *θ*_2_ = 135°). Black dashed circle: cross-section of the optic nerve. Upper right of each illustration: 3D mesh surface plot of the TIEF distribution of the retinal surface projected to the XOZ plane; middle right: MMEA values along the normalized horizontal and vertical lines; lower right: TIEF distribution in the macular area. Normalized MMEAs along horizontal **(B)** and vertical **(C)** lines calculated from **(A)**.

SUPPLEMENTARY FIGURE S2 The normalized MMEA distribution of the retinal surface under different current ratios, with return electrodes fixed at *θ*_2_ = 105° **(A)**, 120° **(B)**, and 145° **(C)**. Current ratios $\alpha_{X}$ and $\alpha_{X}$ = 0.1, 0.3, 0.5, 0.7, and 0.9, respectively. The specific location of the horizontal electrodes was slightly adjusted referred to Table 2 and Figure 6.

SUPPLEMENTARY FIGURE S3 80% intensity contour ranges (dark blue lines) of normalized MMEA on the retinal surface: return electrode fixed at *θ*_2_ = 120° **(A)** and 145° **(B)** under different current ratios ($\alpha_{X}$ and $\alpha_{X}$ increased by 0.1) were projected to the XOZ plane; green boxes: unclosed contour ranges on the retinal surface, red boxes: contour ranges covered optic nerve cross-section.
