# Supplementary figures and images for: Optic nerve-mediated modulation of temporally interfering electric fields for potential targeted retinal disease therapy: a computational modeling study
Source: Front Neurosci. 2024 Dec 23;18:1518488. doi: 10.3389/fnins.2024.1518488 (PMC11701049; doi:10.3389/fnins.2024.1518488)

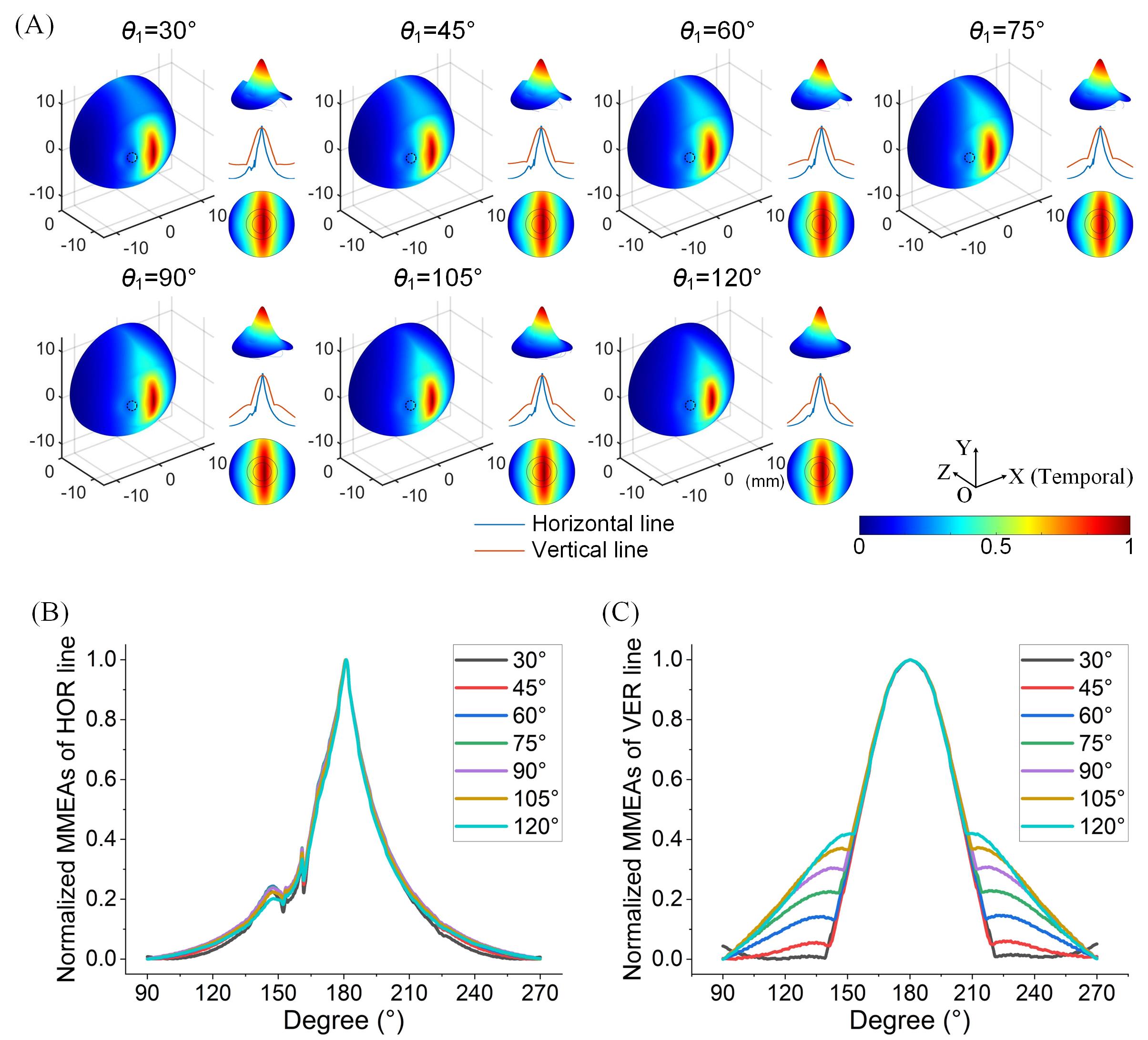

Supplement: SUPPLEMENTARY FIGURE S1 — Spatial distribution of TIEFs under different stimulating electrodes positions. (A) Normalized MMEA distribution of the retinal surface at different return electrodes positions with 2D electrode montage (θ1 = 30°–120°, θ2 = 135°). Black dashed circle: cross-section of the optic nerve. Upper right of each illustration: 3D mesh surface plot of the TIEF distribution of the retinal surface projected to the XOZ plane; middle right: MMEA values along the normalized horizontal and vertical lines; lower right: TIEF distribution in the macular area. Normalized MMEAs along horizontal (B) and vertical (C) lines calculated from (A). [file Image_1.JPEG]

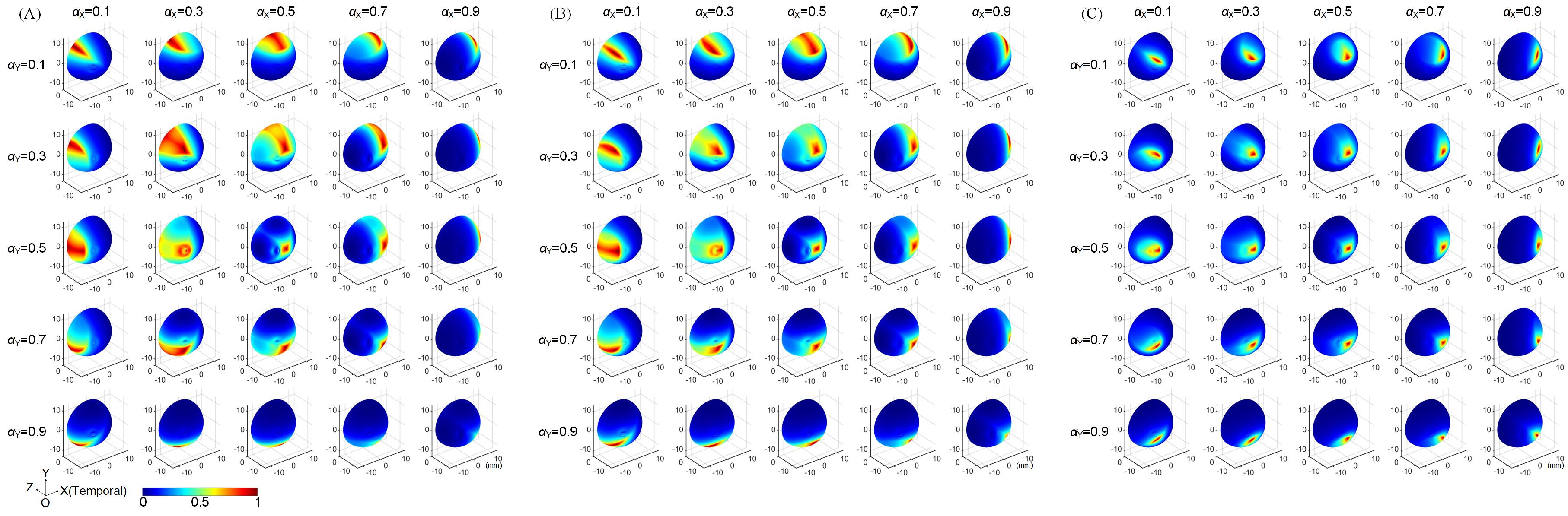

Supplement: SUPPLEMENTARY FIGURE S2 — The normalized MMEA distribution of the retinal surface under different current ratios, with return electrodes fixed at θ2 = 105° (A), 120° (B), and 145° (C). Current ratios αX and αX = 0.1, 0.3, 0.5, 0.7, and 0.9, respectively. The specific location of the horizontal electrodes was slightly adjusted referred to Table 2 and Figure 6. [file Image_2.JPEG]

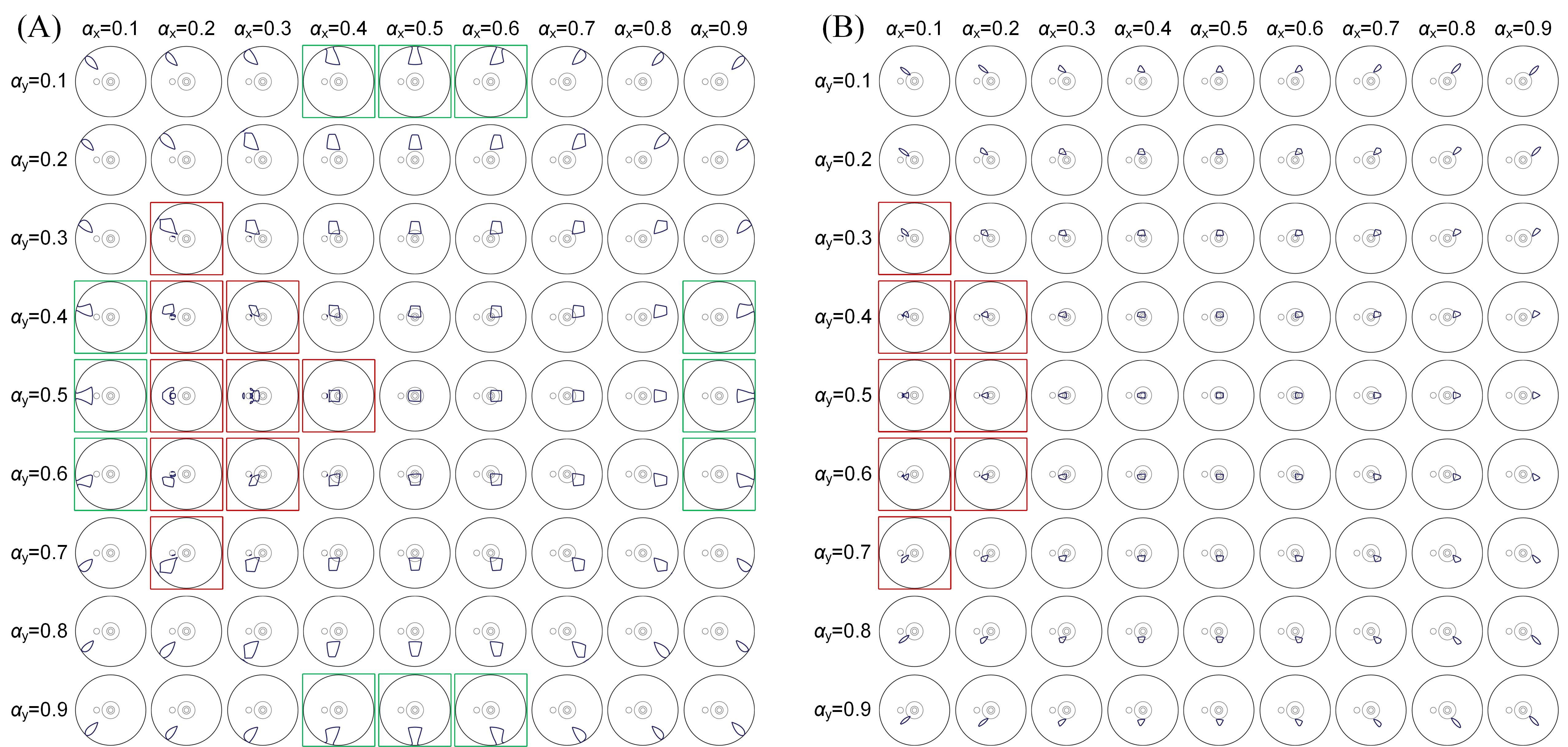

Supplement: SUPPLEMENTARY FIGURE S3 — 80% intensity contour ranges (dark blue lines) of normalized MMEA on the retinal surface: return electrode fixed at θ2 = 120° (A) and 145° (B) under different current ratios (αX and αX increased by 0.1) were projected to the XOZ plane; green boxes: unclosed contour ranges on the retinal surface, red boxes: contour ranges covered optic nerve cross-section. [file Image_3.JPEG]
